# The Natural History and Reperfusion Therapy Outcomes of Acute Ischemic Stroke due to Isolated M2 Occlusions

**DOI:** 10.1155/2021/6626604

**Published:** 2021-04-27

**Authors:** Hongmin Gong, Libo Zhao, Ge Tang, Yu Chen, Deyu Yang, Shudong Liu

**Affiliations:** ^1^Department of Neurology, Yongchuan Hospital of Chongqing Medical University, Chongqing 402160, China; ^2^Chongqing Key Laboratory of Cerebrovascular Disease Research, Chongqing 402160, China

## Abstract

**Objective:**

Currently, the standard treatment modality for patients with acute ischemic stroke (AIS) presenting with isolated M2 occlusions is not specific. We therefore assessed the difference in treatment outcomes for patients with isolated M2 occlusions.

**Methods:**

We retrospectively analyzed consecutive patients with AIS presenting with isolated M2 occlusions from October 1, 2018, to June 30, 2020. Patients were divided into 3 groups based on the treatments they received: no reperfusion therapy (NRT), intravenous thrombolysis treatment (IVT), and endovascular intervention (EVT), which comprised IVT in conjunction with EVT or EVT alone. The primary outcomes were improvements in modified Rankin Scale (mRS) scores at 90 days and National Institutes of Health Stroke Scale (NIHSS) scores at 24 hours after treatment compared with the baseline. The secondary efficacy outcome comprised a good outcome rate defined as a 90 − day mRS score ≤ 2, final infarct volume (FIV), 90-day mortality rate, and successful recanalization rate, which was defined as a modified thrombolysis in cerebral infarction score ≥ 2b. Safety outcomes included symptomatic intracerebral hemorrhage and procedure-related complications.

**Results:**

Seventy patients were enrolled and divided into 3 groups: the NRT group (*n* = 25), IVT group (*n* = 27), and EVT group (*n* = 18). Twenty-four-hour posttreatment NIHSS scores were substantially decreased by EVT compared with NRT (adjusted *β* -4.01, 95% confidence interval [CI] -6.60 to -1.43; *P* = 0.003) or IVT (adjusted *β*, -3.61 [95% CI, -6.45 to -0.77]; *P* = 0.013). Compared with the outcomes observed after NRT, patients who received EVT were more likely to achieve lower 90-day mRS scores (adjusted *β*, -1.42 [95% CI, -2.66 to -0.63]; *P* = 0.007), higher good outcome rates (adjusted odds ratio, 8.73 [95% CI, 1.43-53.24]; *P* = 0.019), and smaller FIVs (adjusted *β*, -29.66 [95% CI, -59.73 to 0.42]; *P* = 0.048). The recanalization rate of EVT was high (88.89%), and procedure-related complications were rare (5.56%).

**Conclusions:**

For acute, isolated M2 occlusions, EVT could dramatically and rapidly improve neurological deficits with high safety and effectiveness. These changes were observed at 24 hours after treatment and were maintained over the long term.

## 1. Introduction

M2 occlusions are an important subgroup of anterior circulation vessel occlusions, accounting for 38.1% [1]. The outcome of patients with M2 occlusions is believed to be good and superior to that of patients with large vessel occlusions (LVOs) in the anterior circulation [2]. Actually, the outcome is much worse if no vascularization therapies are applied [1, 3].

Endovascular intervention (EVT) has become the standard first-line treatment for patients with acute ischemic stroke (AIS) presenting with LVO in the anterior circulation [4–9]. These studies mainly focused on proximal intracranial artery occlusions but largely ignored M2 occlusions. Thus, physicians questioned the efficacy and safety of EVT for patients with distal occlusions. Some analyses subsequently showed that patients with M2 occlusions might benefit from EVT if they met the EVT trial protocols. However, the studies were retrospective analysis and post hoc analysis rather than randomized controlled study, the evidence applied was insufficient [10, 11]. In addition, the treatment methods applied in the control group consisted of conventional medicinal treatment and intravenous tissue plasminogen activator (t-PA), and the favorable response of distal occlusions to t-PA was neglected [12]. Confusion remains regarding the appropriate treatment for M2 occlusions.

We compared the comprehensive presentation, imaging, and clinical outcomes of patients who received no reperfusion therapy (NRT) as a control group with patients who received IVT alone and patients who received EVT, intending to provide evidence to clinical physicians that would enable them to choose an effective and safe treatment for isolated M2 occlusions.

## 2. Subjects and Methods

### 2.1. Subjects

After obtaining institutional review committee approval, we performed a retrospective cohort analysis of patients with AIS who presented with isolated M2 occlusions at the Yongchuan Hospital of Chongqing Medical University from October 1, 2018, to June 30, 2020. The inclusion criteria included time from last normal appearance to first computed tomographic angiography [CTA] of <24 hours, CTA-diagnosed isolated M2 occlusion, >18 years of age, National Institutes of Health Stroke Scale [NIHSS] score at admission ≥ 6 points, and available follow-up data within 90 days. Patients with a premorbid modified Rankin Scale (mRS) score of ≥3 points (*n* = 2), patients who lacked follow-up (within 2-7 days after admission) imaging data (including computed tomography [CT] or magnetic resonance imaging [MRI]) (*n* = 8), or patients whose previous intracranial lesions were affected when calculating the final infarct volume (FIV) due to this stroke (*n* = 2) were excluded. Finally, 70 patients with AIS presenting with isolated M2 occlusions were included and divided into 3 groups: the NRT group (*n* = 25), IVT group (*n* = 27), and EVT group (*n* = 18). Patients who neither met the intravenous thrombolysis criterion of the Chinese Guidelines for the Diagnosis and Treatment of Acute Ischemic Stroke 2018 nor met the indications for EVT in our study and those who met the criterion listed above but refused the two reperfusion treatments were enrolled in the NRT group. All patients in the IVT group met the criterion and agreed to receive IVT. Those who received IVT followed by EVT were included in the EVT group rather than the IVT group. Patients were eligible for EVT if the time from the last normal appearance to femoral artery puncture ≤ 24 hours, preoperative NIHSS score ≥ 6, and RAPID software (iSchemaView) estimated the following CT perfusion (CTP) data: initial infarct volume (ischemic core) < 70 ml, a ratio of the volume of ischemic tissue to initial infarct volume of ≥1.8, and an absolute volume of potentially reversible ischemia (penumbra) of ≥15 ml [8].

All enrolled patients received a head CT scan and CT angiography (CTA) of the head and neck at admission. Once CTA diagnosed M2 occlusions, CTP was essential to estimate the EVT criterion. Within the next 2-7 days after admission, a cranial MRI or CT scan was essential to calculate the final infarct volume (FIV). Information on baseline demographics, vascular risk factors (diabetes mellitus, hypertension, atrial fibrillation, hyperlipidemia, smoking, and congestive heart failure), current medications, including antiplatelet drugs and statins, blood glucose level at admission, time from symptom onset to treatment (the treatment referring to the initial antiplatelet drug treatment in the NRT group, venipuncture in the IVT group, and femoral artery puncture in the EVT group), and hospitalization days were collected from admission and discharge records.

The NIHSS score (range of 0-42 points, with higher scores indicating more serious stroke severity) at admission and at 24 hours posttreatment and the mRS score (range of 0-6 points, with higher scores indicating worse outcomes; a good outcome was defined as a mRS score of 0-2) at admission and at 90 days after symptom onset were assessed by a trained neurological physician who was blinded to the imaging data.

### 2.2. Study Treatment and Intervention

Patients in the NRT group received conservative medicinal treatment. In the IVT group, patients presenting within the first 4.5 hours from the last normal appearance were intravenously administered a full dose (0.9 mg per kilogram) of t-PA. In the EVT group, subjects who received IVT did not undergo heparinization, and a heparin infusion was administered at a dose of 70 U/kg at initiation of the procedure in those who had not received IVT. Before the thrombectomy procedure, three-dimensional digital subtraction angiography (3D-DSA) was performed under local anesthesia to identify the specific occlusion site and the morphology of the M2 segment. A direct first pass aspiration technique (ADAPT) was first considered during the thrombectomy procedure. If the M2 segment failed to be recanalized after three attempts, then a stent retriever was used as a rescue therapy. The devices used included Penumbra 3Max/4Max catheters and SOLITAIRE™ AB retrievers. 3D-DSA was performed again at the end of the operation to evaluate the recanalization status.

### 2.3. Neuroimaging Review

The occlusive site was defined by two authors based on the initial CTA. The beginning of the M2 segment was defined as the vertical segment lying within the mesial margin of the sylvian fissure at the CTA coronal position. Regional leptomeningeal scores (rLMCs, scores ranging from 0-20 points, with higher scores indicating better collateral circulation) were obtained at the coronal and horizontal planes of the initial CTA [13]. FIV was measured on follow-up (within the next 2-7 days after admission) MRI (diffusion-weighted [DWI] sequence) or CT images. We outlined the area of the infarct on each slice using the Mimics software and then summed the individual slice thicknesses of all outlined areas [13]. If the CT or MRI images completed within 7 days after treatment suggested cerebral hemorrhage, it was defined as symptomatic intracerebral hemorrhage (sICH) with an NIHSS score that increased by ≥4 points from baseline; otherwise, it was defined as asymptomatic intracerebral hemorrhage (aICH). For the EVT group, the rate of successful recanalization (defined as a modified thrombolysis in cerebral ischemia [mTICI] score ≥ 2b) and the occurrence of procedure-related complications was determined from postprocedure images of catheter-based angiograms [14]. Two neuroradiologists who were blinded to the group assignments, clinical data, and outcomes independently estimated all neuroimaging findings. The third experienced neuroradiologist was in charge of resolving cases in which a disagreement occurred.

### 2.4. Clinical and Radiological Variables

The delta NIHSS score (change in the NIHSS score from baseline at 24 h) and 90-day modified Rankin Scale score (mRS, range of 0 to 6 points, an excellent outcome was defined as an mRS score of 0-1, a good outcome was defined as an mRS score of 0-2, a bad outcome was defined as a mRS score of 3-5, and a mRS score of 6 referred to death) were the primary efficacy outcomes. The secondary efficacy outcomes included the 90-day good outcome rate (mRS score of 0-2), 90-day mortality rate, FIV, and successful recanalization rate. The safety outcomes included sICH and procedure-related complications.

### 2.5. Statistical Analysis

We compared baseline characteristics and clinical outcomes between the 3 groups. Continuous variables are presented as the means with standard deviations (SD) or medians and interquartile ranges (IQRs), and comparisons were performed using the Kruskal-Wallis test or analysis of variance (ANOVA). Categorical variables are reported as percentages [numbers (%)], and comparisons were performed using Fisher's exact probability method and *χ*^2^ test, as appropriate. The rank sum test was used for grade data. The difference in the NIHSS score between baseline and 24 hours after treatment in each group was investigated using the Mann-Whitney *U* test. We performed two logistic regression analyses, linear regression, and binary logistic regression analyses, to determine the efficacy outcomes. The distributions of 90-day mRS scores, delta NIHSS scores, and FIVs were estimated using linear regression analyses. A 90-day mRS score of 0-2 was estimated using a binary logistic regression analysis. Adjusted estimates of outcome (common odds ratio and *β*) were calculated by considering the following variables: age, baseline NIHSS scores, and time from symptom onset to treatment. A *P* value *<*0.05 was considered significant. All analyses were conducted using the SPSS 23.0 software (IBM SPSS Statistics, Armonk, NY).

## 3. Results

### 3.1. Baseline Characteristics

Seventy patients (36 males [51.43%]; 34 females [48.57%]; mean [SD] age, 74.86 [9.72] years) were included in the analysis, of whom 25 received NRT, 27 received only IVT, and 18 received EVT. Of the 25 patients who did not receive IVT in the NRT group, 16 patients had a long interval from the last known normal appearance to treatment, 3 patients had a large FIV at admission, 1 patient achieved a significant improvement in neurological deficit as soon as the head CT was complete, and the remaining 3 patients met the indications but refused IVT. Of the 18 patients in the EVT group, 11 patients were treated with IVT in conjunction with EVT, as they did not achieve immediate and significant improvement in neurological deficit and the DSA suggested that the M2 segment was still occluded before the endovascular procedure. Another 7 patients were treated with EVT alone. Only 1 of 16 patients who received a direct first pass aspiration technique (ADAPT) failed to achieve successful recanalization after three attempts and then received stent retriever as a rescue therapy; 2 patients only received a stent retriever.

Table 1 shows the baseline characteristics. Patients in the EVT group were younger (EVT vs. NRT, 67.61 [±11.43] vs. 75.16 [±7.60] years, *P* = 0.065; EVT vs. IVT, 67.61 [±11.43] vs. 79.41 [±7.36] years, *P* < 0.001). The time from symptom onset to treatment of patients in the IVT group (154 [125.0, 238.0], min) was shorter than that in the NRT group (370 [230.5, 820.5], min; *P* < 0.001) and the EVT group (272.5 [183.8, 386.3], min; *P* < 0.001) (Table 2). Otherwise, no significant differences were observed in sex, rLMCs, vascular risk factors, drugs currently used, admission serum glucose levels and systolic blood pressure, or hospitalization days.

### 3.2. Clinical Outcomes

**Figure 1:**
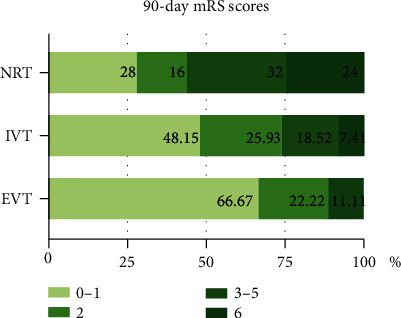
Distribution of mRS scores at 90 days in the three groups. Notes: proportion of mRS scores at 90 days in patients in the NRT, IVT, and EVT groups. NRT: no reperfusion therapy; IVT: intravenous thrombolysis treatment; EVT: endovascular intervention; mRS: modified Rankin Scale. A 90-day mRS score of 0-1 was defined as an excellent outcome, an mRS score of 0-2 was defined as a good outcome, an mRS score of 3-5 was defined as a bad outcome, and an mRS score of 6 referred to death.

**Figure 2:**
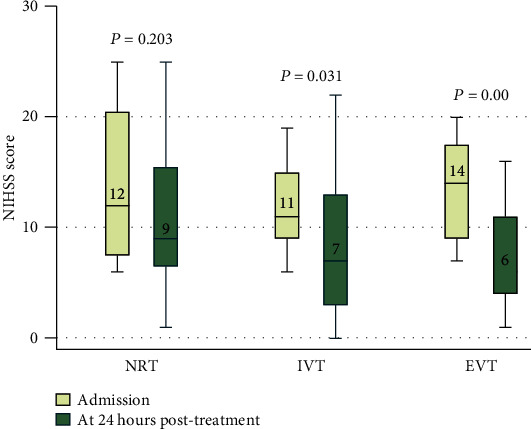
Comparison of NIHSS scores among the three groups. Notes: box and whisker plots comparing median NIHSS scores at admission and 24 hours after treatment in the no reperfusion therapy (NRT), intravenous thrombolysis treatment (IVT), and endovascular intervention (EVT) groups. The horizontal line in the middle of each box represents the median, and the upper and lower boundaries of the box represent the 75th percentile and 25th percentile, respectively. The whiskers above and below the box indicate the 90th and 10th percentiles, respectively.

Table 3 shows the efficacy and safety outcomes. The median 90-day mRS score was 4 (IQR, 1-5) in the NRT group, 2 (IQR, 1-3) in the IVT group, and 1 (IQR, 1-2) in the EVT group. A trend toward lower disability grades was observed in patients who received reperfusion treatments (unadjusted *β* values of -1.13 and -1.64, respectively; *P* values were all <0.05) (Table 3 and Figure 1). However, after adjustment for age, the NIHSS score at admission, and time from onset to treatment, we only showed a significant difference favoring EVT, with an adjusted *β* value of -1.42 (95% confidence interval [CI], -2.66 to -0.63; *P* = 0.007). The most dramatic difference in the NIHSS score at 24 hours after treatment from baseline was detected in the EVT group (median NIHSS score of 6 vs. 14; *P* < 0.001) (Figure 2). Compared with the NRT group (adjusted *β* -4.01 [95% CI, -6.60 to -1.43], *P* = 0.003) and the IVT group (adjusted *β* -3.61 [95% CI, -6.45 to -0.77], *P* = 0.013), the value was greater in the EVT group. Compared with the NRT group, the 90-day good outcome (mRS 0-2) rate (88.89% vs. 44.00%] adjusted OR 8.73 [95% CI, 1.43-53.24; *P* = 0.019]) was higher and the FIV within 7 days after treatment (50.93 [8.55, 112.75] vs. 26.08 [6.78, 53.63]; adjusted *β* -29.66 [95% CI, -59.73 to 0.42]; *P* = 0.048) was smaller in the EVT group. The recanalization rate in the EVT group was high (88.89%). The 90-day mortality rate in the NRT group was higher than that in the other 2 groups (NRT vs. IVT vs. EVT, 24% vs. 7.41% vs. 0%).

Regarding safety outcomes, patients in the IVT group were more vulnerable to sICH (NRT vs. IVT vs. EVT, 0% vs. 14.81% vs. 0%) (Table 3). Only 1 of the 18 patients who received EVT (5.56%) had a procedure-related complication, namely, vessel perforation. The M2 segment was perforated when the operator delivered the 3Max catheter to the terminus of the M1 segment and intended to move the catheter toward the occluded M2 segment. After stopping the superselection and starting digital subtraction angiography (DSA), contrast was extravasating from the perforated vessel, the operator terminated the operation immediately, and M2 therefore failed to be recanalized. The postprocedure CT of this patient showed minor subarachnoid hemorrhage, and the neurological deficit did not deteriorate, whereas the 90-day mRS score was 5.

## 4. Discussion

This retrospective, observational study analyzed consecutive patients who presented with acute symptomatic M2 occlusions. In our study, only 44% (11/25) of patients who received NRT achieved good outcomes at 90 days, and the mortality rate at 90 days reached 24% (6/25), similar to the results reported previously, where the 90-day good outcome rate ranged from 45.8 to 47.3% and the mortality rate at 6 months reached 20.8% [1, 3]. This finding revealed the severely poor natural history of patients with isolated M2 occlusions. Patients who received reperfusion treatment, either IVT or EVT, had a tendency toward achieving good functional outcomes at 90 days. In particular, patients treated with EVT were more likely to achieve an improvement in neurological deficits with high effectiveness and safety, which was observed at 24 hours after treatment and was maintained over the long term.

Our study suggested that the FIV of patients who received EVT was significantly smaller and that the 90-day functional outcomes were better than those of patients who received NRT. A smaller infarct volume was indicated to be associated with better functional outcomes [15]. Thus, the effect of EVT on functional outcome was possibly mediated by decreasing the FIV. However, the study was underpowered to draw the conclusion, as no causal mediation model was constructed in our study. As shown in the study by Compagne et al., preventing FIV progression only partially explains the beneficial effect of EVT on outcomes using mediation analysis [16]. The reperfusion of some key regions, such as the lateral fissure in the dominant hemisphere and the M3 and M6 regions in the Alberta Stroke Program Early CT Score, probably played a crucial role in favorable functional outcomes [17].

In our analysis, the change in NIHSS score from baseline to 24 hours after treatment in the IVT group was significant, and most patients (20/27, 74.07%) achieved good 90-day functional outcomes. Distal intracranial vessel occlusions responded well to t-PA with a high recanalization rate of 30.8–68.4%, which is strongly associated with a 90-day good outcome, even when it occurs two hours after the t-PA bolus [18–22]. We therefore hypothesized that some patients who received only IVT had achieved successful recanalization after treatment. However, we were unable to determine whether the M2 segment recanalized due to the lack of follow-up vascular imaging. Furthermore, as the process of recanalization with intravenous t-PA was continuous over time, the time from t-PA bolus administration to recanalization assessment was associated with successful recanalization (odds ratio, 1.28 for every 30-minute increase in time [95% CI, 1.18-1.38]) [19]. This finding would raise concern about the time within which the follow-up vascular imaging should be completed after the administration of the t-PA bolus and the time at which the vessel recanalized would contribute to good outcomes.

Evidence has shown the benefit of EVT for M2 segment occlusions compared with the best medical treatment or M1 segment [11, 23]. Moreover, the 2 mechanical thrombectomy techniques most frequently utilized to treat M2 occlusions, ADAPT and stent retriever, did not differ in rates of 90-day good outcomes, sICH, successful recanalization, or mortality [24, 25]. In our study, the successful recanalization rate of patients who mainly received ADAPT reached 88.89% without sICH occurrence. In previous studies, the successful recanalization rate in the ADAPT group was lower (70.3-85.4%), and the sICH rate was higher (5.3-5.6%) [24, 25]. We hypothesized that the prioritization of effectiveness and safety in our study might be associated with the 3D-DSA used in the process of operation, which is often used in the diagnosis and treatment of cerebral aneurysms but is rarely mentioned in the process of EVT [26–28]. The M2 segment usually consists of 6-8 branches, and each branch has a different morphology and diameter, resulting in overlapping images on traditional two-dimensional digital subtraction angiography (2D-DSA), which would increase the operational difficulty and decrease the possibility of recanalization. 3D-DSA is helpful to observe the occluded M2 segment at some angles and planes that were missed using 2D-DSA. Further, random controlled trial to compare only EVT with EVT plus 3D-DSA in patients with acute M2 occlusions is probably a priority.

In our study, the 90-day good outcome rates in the IVT group and EVT group both were high (74.07% vs. 88.89%) and the difference between the two groups was not significant (*P* = 0.186). Patients received reperfusion treatment, either IVT or EVT, had a tendency toward achieving 90-day good functional outcomes. But on the other hand, the ability of EVT to decrease the NIHSS score at 24 hours after treatment from baseline was more significant than IVT (adjusted *β* -3.61 (-6.45 to -0.77); *P* = 0.013). Therefore, we recommend that EVT should be performed when a neurological improvement is not apparent after IVT.

Our study has several limitations. First, our study is a post hoc analysis and has all the limitations of a nonrandomized study. Second, we enrolled a relatively small number of patients, especially patients who received EVT, resulting in poorly balanced NIHSS score strata and the lack of stratification of patients in the EVT group for IVT. Third, follow-up vascular images captured after treatment were lacking in the NRT and IVT groups, thus leaving a gap in our understanding of the relationship between clinical recovery and successful reperfusion. Fourth, the outcomes of patients in different NIHSS score strata were not analyzed, and we were unable to recommend the NIHSS score strata within which IVT or EVT should be performed.

In summary, the natural history of patients with isolated M2 occlusions was very poor. The EVT modality dramatically and rapidly improved the clinical outcomes with higher safety and effectiveness, and the changes were present at 24 hours after treatment and maintained over the long term.

## Figures and Tables

**Table 1:** Baseline patient characteristics.

Characteristics	NRT (*n* = 25)	IVT (*n* = 27)	EVT (*n* = 18)	*F*/*Z*/*χ*2	*P* value
Age, mean ± SD, years	75.16 ± 7.60	79.41 ± 7.36	67.61 ± 11.43	12.42^a^	<0.01
Male (%)	13 (52)	12 (44.44)	11 (61.11)	1.21^b^	0.547
Left hemisphere (%)	17 (68)	15 (55.56)	13 (72.22)	1.54^b^	0.463
rLMCs, median (IQR)	15 (12, 17)	17 (14.7, 19)	16 (14, 18)	4.76^a^	0.105
Admission NIHSS score, median (IQR)	12 (7.5, 20.5)	11 (9, 15)	14 (9, 17.5)	2.42^a^	0.298
Admission NIHSS score stratification (%)					
<13	13 (52)	18 (66.67)	8 (44.44)	4.70^c^	0.095
13-19	5 (20)	9 (33.33)	9 (50)		
≥ 20	7 (28)	0	1 (5.56)		
Vascular risk factors					
Hypertension (%)	15 (60)	11 (40.74)	5 (27.78)	4.63^b^	0.099
Diabetes (%)	4 (16)	6 (22.22)	5 (27.78)	0.88^b^	0.644
Hyperlipidemia (%)	8 (32)	3 (11.11)	7 (38.89)	5.17^b^	0.076
Coronary heart disease (%)	3 (12)	8 (29.63)	4 (22.22)	2.41^b^	0.30
Congestive heart failure (%)	5 (20)	2 (7.41)	5 (27.78)	3.47^d^	0.171
Atrial fibrillation (%)	8 (32)	8 (29.63)	10 (55.56)	3.55^b^	0.186
History of ischemic stroke (%)	4 (16)	4 (14.81)	1 (5.56)	1.13^d^	0.668
Drinking (%)	1 (4)	2 (7.41)	3 (16.67)	2.08^d^	0.428
Smoking (%)	4 (16)	3 (11.11)	6 (33.33)	3.42^d^	0.166
Antiplatelet drugs are currently used (%)	1 (4)	0	3 (16.67)	4.62^d^	0.059
Statins are currently used (%)	1 (4)	0	3 (16.67)	4.62^d^	0.059
Admission serum glucose level, mean ± SD, mmol/l	8.06 ± 4.72	7.58 ± 2.39	7.71 ± 2.43	0.42^a^	0.81
Admission systolic blood pressure, mean ± SD, mmHg	156.8 ± 25.7	154 ± 22.06	142.67 ± 18.77	2.19^e^	0.12
Hospitalization days, mean ± SD	13.04 ± 4.95	11.70 ± 5.04	11.06 ± 4.54	0.95^e^	0.39
Time from onset to treatment, median (IQR), min	370 (230.5, 820.5)	154 (125.0, 238.0)	272.5 (183.8, 386.3)	20.80^a^	<0.01

Abbreviations: NRT: no reperfusion therapy; IVT: intravenous thrombolysis treatment; EVT: endovascular intervention; rLMCs: regional leptomeningeal score; NIHSS: National Institutes of Health Stroke Scale; SD: standard deviation; IQR: interquartile range. ^a^Kruskal-Wallis test; ^b^*χ*^2^ test; ^c^rank sum test; ^d^Fisher's exact probability method; ^e^ANOVA.

**Table 2:** Multiple comparisons based on age and time from onset to treatment.

Characteristics	NRT vs. IVT	NRT vs. EVT	IVT vs. EVT
Age, years	0.073^∗^	0.065^∗^	<0.001^∗^
Time from onset to treatment, min	<0.001^∗^	0.279^∗^	<0.001^∗^

^∗^ *P* value.

**Table 3:** Efficacy and safety outcomes according to treatment methods.

Characteristic	NRT	IVT	EVT	Unadjusted value (95% CI); *P* value	Adjusted value (95% CI)^†^: *P* value
Primary efficacy outcome					
Delta NIHSS score, median (IQR)	-1 (-2.5 to 0)	-3 (-6 to 0)	-5 (-10.25 to -3.75)	-0.98 (-3.18 to 1.22); *P* = 0.38^∗^ -4.26 (-6.71 to -1.81); *P* = 0.001^#^ -3.28 (-5.69 to -0.87); *P* = 0.008^▲^	-0.40 (-2.89 to 2.09); *P* = 0.750^∗^ -4.01(-6.60 to -1.43); *P* = 0.003^#^ -3.61 (-6.45 to -0.77); *P* = 0.013^▲^
90-day mRS score, median (IQR)	4 (1, 5)	2 (1, 3)	1 (1, 2)	-1.13 (-2.04 to -0.22); *P* = 0.016^∗^ -1.64 (-2.66 to -0.63); *P* = 0.002^#^ -0.52 (-1.52 to 0.48); *P* = 0.303^▲^	-0.67 (-1.65 to 0.32); *P* = 0.181^∗^ -1.42 (-2.43 to -0.40); *P* = 0.007^#^ -0.75 (-1.87 to 0.37); *P* = 0.185^▲^
Secondary efficacy outcomes					
90-day mRS score 0-2 (%)	11 (44.00)	20 (74.07)	16 (88.89)	3.64 (1.13-11.69); *P* = 0.03^∗^ 10.18 (1.92-54.02); *P* = 0.006^#^ 2.80 (0.51-15.38); *P* = 0.236^▲^	2.23 (0.51-9.64); *P* = 0.284^∗^ 8.73 (1.43-53.24); *P* = 0.019^#^ 6.38 (0.41-99.12); *P* = 0.186^▲^
FIV, median (IQR), ml	50.93 (8.55, 112.75)	30.77 (8.75, 55.270)	26.08 (6.78, 53.63)	-43.28 (-72.97 to -13.59); *P* = 0.005^∗^ -37.24 (-70.32 to -4.17); *P* = 0.028^#^ 6.04(-26.52 to 38.59); *P* = 0.713^▲^	-8.63 (-37.67 to 20.41); *P* = 0.555^∗^ -29.66 (-59.73 to 0.42); *P* = 0.048^#^ -21.03 (-54.12 to 12.07); *P* = 0.209^▲^
90-day mortality rate (%)	6 (24)	2 (7.41)	0	NA	NA
mTICI ≥ 2b (%)	-	-	16 (88.89)	NA	NA
Safety outcomes					
sICH (%)	0	4 (14.81)	0	NA	NA
Procedure-related complications (%)	-	-	1 (5.56)	NA	NA

Abbreviations: NRT: no reperfusion therapy; IVT: intravenous thrombolysis treatment; EVT: endovascular intervention; NIHSS: National Institutes of Health Stroke Scale; IQR: interquartile range; mRS: modified Rankin Scale; sICH: symptomatic intracerebral hemorrhage; aICH: asymptomatic intracerebral hemorrhage; mTICI: modified thrombolysis in cerebral ischemia score; FIV: final infarct volume (within 2-7 days after symptom onset); NA: not applicable; delta NIHSS score: change in the NIHSS score from baseline to 24 h. ^†^Model adjusted for age, admission NIHSS score, and time from onset to treatment. ^∗^A comparison was conducted between the NRT and IVT groups, and ^#^comparison conducted between the NRT and EVT groups. ^▲^A comparison was conducted between the IVT and EVT groups.

## Data Availability

If necessary, we can provide the crude data via the E-mail of corresponding author.
